# Heat Transfer Enhancement in the Microscale: Optimization of Fluid Flow

**DOI:** 10.3390/nano12203628

**Published:** 2022-10-16

**Authors:** Joshua Beck, Michael Palmer, Kallie Inman, Jake Wohld, Marcus Cummings, Ryan Fulmer, Branden Scherer, Saeid Vafaei

**Affiliations:** Mechanical Engineering Department, Bradley University, Peoria, IL 61606, USA

**Keywords:** nanoparticles, microchannels, converging connector, spherical connector, heat transfer coefficient, thermal conductivity, viscosity

## Abstract

The focus of this paper is to investigate the effects of the addition of a connector between two serial microchannels. The idea of adding connector at the inlet of microchannels to enhance the random motion of molecules or nanoparticles in low Reynolds numbers was developed in our research group for the first time. It was experimentally determined that the shape of a connector between two microchannels has a significant impact on the enhancement of the random motion of molecules or nanoparticles. Consequently, the heat transfer coefficient is improved inside the second microchannel. The connector is large enough to refresh the memory of the fluid before entering the second channel, causing a higher maximum heat transfer coefficient in the second channel. It was also observed that the heat transfer coefficient can be increased at the end of the channel when the outlet temperature is relatively high. This may be explained by the fact that as temperature increases, the fluid viscosity tends to decrease, which generally drives an increase in the local random motion of base fluid molecules and nanoparticles. This causes an increase in the microchannel heat transfer coefficient. It was found that the addition of nanoparticles significantly modified the impact of the connector on the microchannel heat transfer coefficient. In addition, the effects of changing the Reynolds number and the shape of the connector were investigated through use of computational fluid dynamics (CFD) calculations. It was found that both factors have an important impact on the variation of velocity and enhancement of random motion of molecules and consequently significantly affect the heat transfer coefficient.

## 1. Introduction

As technology continues to advance, the required rate of power dissipation in modern systems grows. In order to meet this demand, the need for effective heat transfer within systems is increasing. At the same time, systems are shrinking in size, particularly in the electrical industry. As such, it is imperative that the cooling systems used are both more effective and smaller in scale. Therefore, traditional methods of thermal management are becoming less sufficient for use in modern systems. Many traditional yet effective cooling systems have utilized working fluids such as water, ethylene glycol, propylene glycol, or oil to transport heat away from critical power dissipating components. When considering new methods of thermal management, techniques for further improving fluid cooling have been examined.

Xuan and Li [1] proposed theoretical mechanisms by which the addition of nanoparticles in a fluid can enhance the convective heat transfer coefficient of the fluid. They suggested that aside from thermal conductivity enhancement, the general heat transfer of a nanofluid may be improved due to its increased surface area and heat capacity. Additionally, the solid particles may cause increased mixing and higher turbulence in the fluid, leading to improved energy transfer between fluid layers. It was also found that modifying the concentration [2], shape [2], size [3,4], and flow regime [5] of a nanofluid may affect the heat transfer coefficient.

The goal of this paper is to briefly review the effects of nanoparticle concentration and characteristics on the heat transfer properties of a nanofluid. Moreover, it seeks to discuss the optimization of both operating conditions and characteristics of a nanofluid in order to maximize the heat transfer coefficient. This paper will also examine the effects of adding a connector with larger cross-sectional area between two serial microchannels and discuss how the shape of the connector can affect the heat transfer coefficient. Different applications have been modelled through use of CFD calculations, which solve the continuity and Navier–Stokes equations numerically, a technique which has been used for applications in bio-convection [6], modeling magnetohydrodynamic flow [7,8,9], and hybrid nanofluids [10]. The new technique can be applied for thermal management of electronic devices on earth and space. This method will (a) pave a road for efficient thermal management in a wide range of applications and (b) reduce the size of heating systems and enhance the thermal management, simultaneously. The light, miniaturized, and efficient thermal management plays a significant role in space technology [11] the Mars mission [12], and NASA programs [13]

## 2. Brief Review on Optimization of Effects of Nanoparticles

The experiment described in this paper seeks to maximize the heat transfer coefficient in microchannels. The heat transfer coefficient can be largely dependent on the thermal conductivity as well as the viscosity of the working fluid. For both thermal conductivity and viscosity, various nanoparticle and environmental parameters must be considered, including nanoparticle material, nanoparticle concentration, choice of base liquid, and fluid temperature. This section provides a review on these properties’ effects on the thermal conductivity, viscosity, and heat transfer coefficient of nanofluids.

### 2.1. Effects of Nanoparticles on Thermal Conductivity

**Figure 1:**
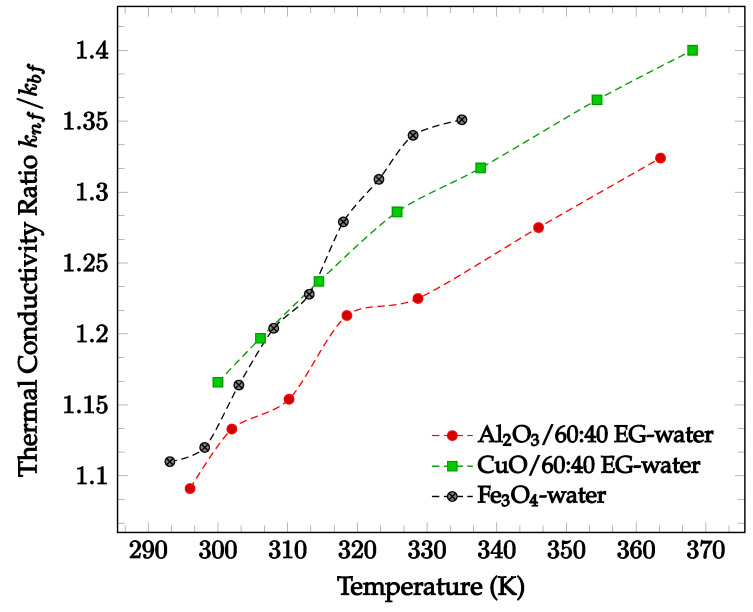
Dimensionless nanofluid thermal conductivity as a function of temperature for different nanofluids at 2% volume concentration, including Al_2_O_3_ in 60–40% ethylene glycol-water [14], CuO in 60–40% ethylene glycol-water [14], and Fe_3_O_4_ in water [15].

In general, the addition of nanoparticles to a base fluid tends to increase its thermal conductivity. This is because the thermal conductivities of the materials used for nanoparticles are generally much higher than the conductivities of base fluids. In addition, random motion of nanoparticles enhances the energy transport from one layer to another. The amount by which thermal conductivity of a fluid is enhanced by the addition of nanoparticles is shown in Figure 1 below. In Figure 1, nanofluids composed of 60:40% ethylene glycol (EG)-water with various nanoparticles are compared. As can be seen, each of the nanofluids exhibits higher thermal conductivities than the base fluid itself. From the data collected, there is no visible correlation between particle thermal conductivity and nanofluid thermal conductivity. Nanofluids using particles with higher conductivity, such as Al_2_O_3_ (k = 36 W/m·K [14]), do not always produce a higher nanofluid thermal conductivity than those using particles with lower conductivity, such as ZnO (k = 13 W/m·K [14]). However, most data demonstrates that thermal conductivity increases with temperature. This increase in thermal conductivity with temperature is driven by the increase in particle motion at high temperatures. As the motion of the particles increases, collisions occur at a more frequent rate, increasing the rate of heat transfer between particles. All four data sets shown in Figure 1 below demonstrate a significant increase in relative thermal conductivity as temperature increases. Since Figure 1 presents the ratio of nanofluid thermal conductivity to base fluid thermal conductivity, the changing thermal conductivity accounts for the fluctuation in base fluid thermal conductivity. As such, the observed increase in nanofluid thermal conductivity may be due to the addition of nanoparticles.

**Figure 2:**
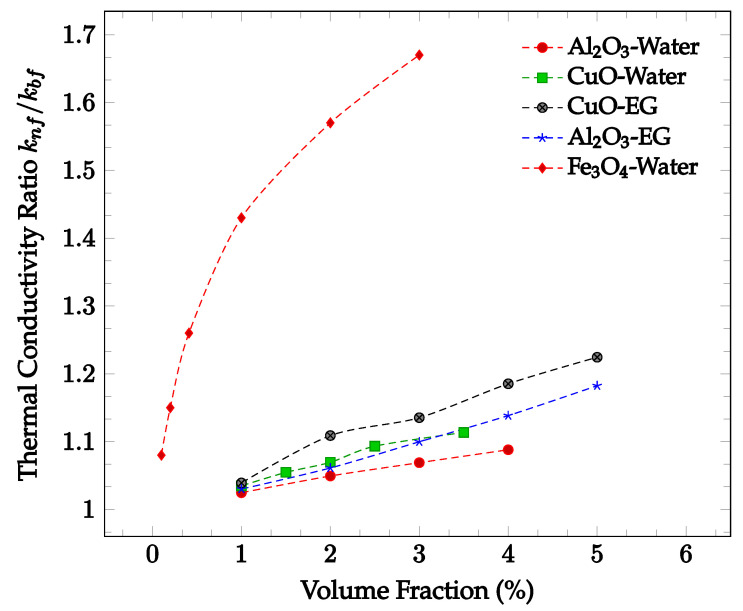
Dimensionless nanofluid thermal conductivity as a function of volume fraction at approximately 300 K for Al_2_O_3_ in water [16], Al_2_O_3_ in ethylene glycol (EG) [17], CuO in water [16], CuO in ethylene glycol (EG) [18], and Fe_3_O_4_ in water [19].

The enhancement of thermal conductivity in nanofluids is also a function of the concentration of nanoparticles. Increasing the volume fraction of nanoparticles tends to increase the thermal conductivity of a fluid. This is likely due to the increased number of collisions and therefore increased random particle motion present in the fluid. Figure 2 below shows thermal conductivity enhancement in CuO and Al_2_O_3_ nanoparticles within water and ethylene-glycol as a function of volume fraction. In all four cases, the thermal conductivity enhancement increases along with nanoparticle concentration. The increase in thermal conductivity with increased concentration may be due to the increase in Brownian motion and particle interactions. The amount of random particle motion is higher due to a greater number of particles in a given volume of fluid. Additionally, as the concentration increases, the likelihood of particle collision increases, enhancing the average rate of energy transfer and thus improving the thermal conductivity of the nanofluid. It can be seen in most samples observed that the greatest rate of thermal conductivity enhancement occurs at lower concentrations. The decrease in the rate of thermal conductivity enhancement may be caused by the tendency of the nanoparticles to agglomerate at higher concentrations. The agglomerates are much larger than the nanoparticles, reducing the effectiveness of the thermal conductivity enhancement. However, the increase in particle concentration still leads to overall thermal conductivity enhancement. As such, the rate of thermal conductivity enhancement is generally highest for lower volume fractions.

The choice of base fluid and addition of a surfactant also affects the thermal conductivity of a nanofluid. It has been found in studies that choosing a base fluid with a higher thermal conductivity may increase the thermal conductivity of the nanofluid. Liu et al. [20] compared the thermal conductivity of carbon nanotube nanofluids using ethylene glycol and engine oil. They found that the average thermal conductivity enhancement ratio using the engine oil was higher than the enhancement ratio of the ethylene glycol nanofluid relative to the base fluids. Agarwal et al. [21] similarly experimented on nanofluids using engine oil and ethylene glycol as base fluids, but with CuO nanoparticles. Conversely, they found that the thermal conductivity enhancement of ethylene glycol nanofluids was generally higher than that of engine oil nanofluids. Barbés et al. [22] compared the thermal conductivities of water and ethylene glycol-based nanofluids using CuO nanoparticles, finding that the water-based nanofluids tended to have significantly higher thermal conductivities than ethylene glycol-based nanofluids for the same concentration. This may be due to the higher thermal conductivity of water than ethylene glycol at a given temperature. To investigate the effect of hybrid base fluids on nanofluid thermal conductivity, Sundar et al. [23] compared nanodiamond nanofluids in different dilutions of ethylene glycol in water. They discovered that nanofluids consisting of a higher ratio of water to ethylene glycol tended to have higher thermal conductivities, as the overall base fluid thermal conductivity was higher [23]. Additionally, it has been found that for both pure liquids and nanofluids, the addition of a surfactant increases the thermal conductivity up to a point, but eventually is lowered as the concentration of surfactant increases. According to Cakmak [24], surfactants are likely to allow nanoparticles to disperse in a fluid more evenly, increasing the nanofluid’s thermal conductivity. However, once the concentration gets high enough, the particles may agglomerate together and fall out of suspension, lowering the overall thermal conductivity. Cakmak [24] verified these theories using graphene oxide (GO)-water nanofluids with sodium dodecyl sulfate (SDS) and triton X-100 (TX 100) surfactants. He found that both types of surfactants increased the average thermal conductivity at 20 °C for surfactant mass concentrations of up to 0.1%. For nanofluid with GO-SDS surfactant, Cakmak [24] found that the ratio of nanofluid thermal conductivity to pure water thermal conductivity was 1.017, and the GO-TX 100 average thermal conductivity ratio compared to pure water was found to be 1.009. In other words, in the given conditions, Cakmak [24] observed an average thermal conductivity enhancement of 1.7% for GO-SDS nanofluid and an enhancement of 0.9% for GO-TX 100 nanofluid relative to pure water. However, at a surfactant mass concentration of 0.2%, both nanofluids were found to have a lower thermal conductivity than that of pure water at the same temperature [24]. This phenomenon was additionally observed by Li et al. [25], who experimented using copper in water with a sodium dodecylbenzene sulfonate surfactant. When this surfactant was used in both the nanofluid and in water without any copper nanoparticles, the average thermal conductivity was found to increase until approximately 0.02% concentration by mass. At this point, the water had roughly a 3.5% increase in average thermal conductivity relative to pure water at the same operating conditions, and the nanofluid saw a 12% increase in average thermal conductivity in comparison to pure water at the same temperature [25]. As the mass concentration of surfactant increased further to 0.12%, the ratio of average nanofluid thermal conductivity to the thermal conductivity of pure water dropped to 1.012, and the average thermal conductivity of the water-surfactant mixture dropped beneath that of pure water [25]. This shows that in order to optimize the thermal conductivity of a nanofluid, a small amount of surfactant may yield higher thermal conductivities, but that the amount must be optimized based on the type of nanofluid.

Li et al. [26] reviewed the effects of nanoparticle shape on the nanofluid thermal conductivity and found that increasing the aspect ratio of rod-shaped particles typically led to significantly higher nanofluid thermal conductivities. In comparison, polygonal nanoparticles typically had lower enhancement levels similar to those of spherical nanoparticles [26]. It was typically found that increasing the size of the nanoparticles led to a decrease in thermal conductivity. Chon et al. [27] compared various sizes of Al_2_O_3_ nanoparticles in water and found that the nanofluids with smaller nanoparticles had a higher thermal conductivity. This may be due to the higher ratio of surface area to volume and increased random motion within the fluid. However, Kwek et al. [28] also investigated the effect of changing nanoparticle size on Al_2_O_3_ nanoparticles in water and found that the thermal conductivity can sometimes increase with increasing nanoparticle size, which may be caused by an increase in diffusive heat transfer. The effects of nanoparticles size were explained in reference [29] in detail.

### 2.2. Effects of Nanoparticles on Viscosity

**Figure 3:**
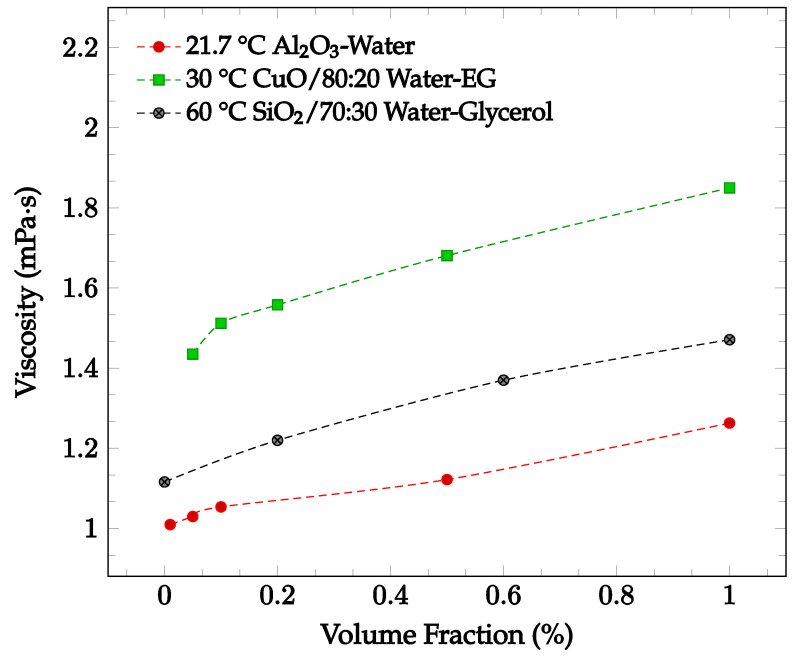
Nanofluid viscosity as a function of concentration for various nanofluids, including Al_2_O_3_ in water at 21.7 °C [30], CuO in 80:20 water-ethylene glycol (EG)at 30 °C [31], and SiO_2_ in 70:30 water-glycerol at 60 °C [32].

The viscosity of a nanofluid may change depending on nanoparticle material, nanoparticle concentration, base fluid, fluid temperature, and addition of a surfactant. It has been found that increasing particle concentration may lead to an increase in nanofluid viscosity. Sekhar and Sharma [30] conducted an experimental study examining the effect of nanoparticle concentration on viscosity for a water-based Al_2_O_3_ nanofluid with volume fractions of 0.01%, 0.05%, 0.1%, 0.5% and 1.0%. The nanofluid viscosity was recorded between temperatures of 25 °C and 45 °C. For the entire temperature range, the viscosity was greatest at the highest concentration of 1.0%. For a temperature of 21.7 °C, the viscosity increases by just over 25% when increasing from the lowest concentration of 0.01% to 1.0% [30], as shown in Figure 3. This relationship could be due to the increased attractions and interactions between the nanoparticles of a higher concentration. The relationship between nanoparticle concentration and nanofluid viscosity was also explored by Esfe and Esfandeh [31] with CuO nanoparticles in a 20:80 EG-water base fluid. The nanofluid viscosity with nanoparticle volume concentrations of 0.05%, 0.1%, 0.2%, 0.5%, and 1.0% were measured over a 15 °C to 50 °C temperature range. As shown in Figure 3, the viscosity increased as the concentration was increased from 0.05% to 1.0%. Lahari et al. [32] studied the effect of the concentration of SiO_2_ nanoparticles in a 70:30 water-glycerol base fluid at temperatures between 20 °C to 80 °C. The viscosity of the nanofluid with concentrations of 0.2%, 0.6%, and 1.0% was found to increase over the entire tested temperature range as the concentration increased. At 60 °C, the average viscosity of the nanofluid when compared to the base fluid as measured alone increased by 9.3%, 22.8%, 31.8% for the concentrations of 0.2%, 0.6%, and 1.0%, respectively [32], as shown in Figure 3.

**Figure 4:**
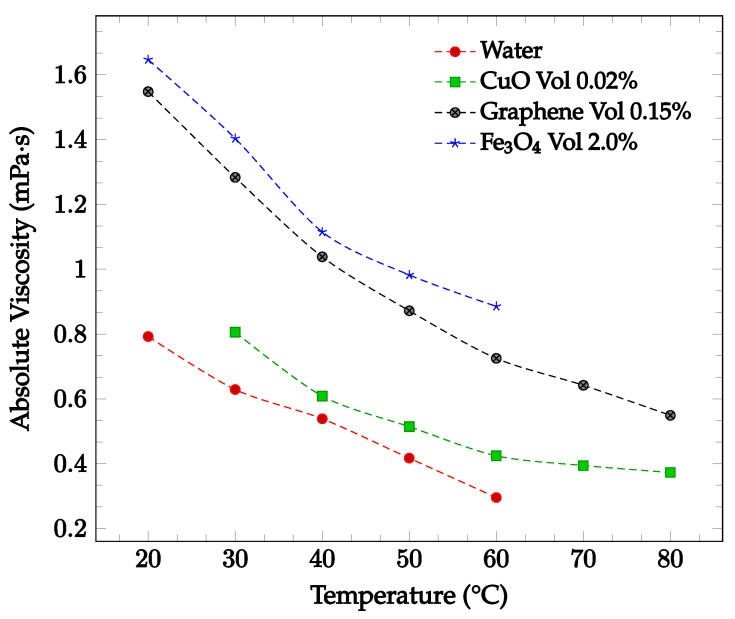
Nanofluid viscosity as a function of temperature for water-based fluids of 0.02% by weight CuO [34], 0.15% by volume graphene [35], and 2.0% by volume Fe_3_O_4_ [15].

The viscosity of a nanofluid can also be observed to decrease as the temperature of the nanofluid increases. This may be due to the weakening of intermolecular forces within the nanofluid as temperature rises, which consequently causes instability and reduces the viscosity of the fluid [33]. Sharma et al. [34] captured the effect of temperature on viscosity for a CuO-water nanofluid between 30 °C to 80 °C with concentrations of 0.02 wt% and 0.1 wt%. As the temperature of the nanofluid increased, the viscosity decreased for both concentrations examined. Furthermore, Ahammed et al. [35] measured the viscosity of graphene-water nanofluid at 0.05%, 0.1%, and 0.15% from 20 °C to 80 °C and concluded that the viscosity of the nanofluid tends to decrease with increasing temperature. This could be due to the intermolecular forces within the nanofluid weakening as the temperature increases. For the 0.15% concentration, the viscosity as a function of temperature is displayed in Figure 4. The effect of temperature on nanofluid viscosity was also studied by Sundar et al. [15] for Fe_3_O_4_-water nanofluid. The experiment was conducted for the base liquid and concentrations up to 2.0%. and temperatures ranging from 20 °C to 60 °C. The viscosity of the nanofluid was again found to decrease as the temperature increased. For the 2.0% concentration nanofluid, the measured viscosity over the studied temperature range is shown in Figure 4. Over the temperature range considered, the viscosity was calculated to decrease by approximately 46%. In each of the studies conducted by Sharma et al. [34], Ahammed et al. [35], and Sundar et al. [15], the effect of increasing viscosity with increasing nanoparticle concentration was also observed.

From Figure 3 and Figure 4, it can be concluded that the nanoparticle material also affects the viscosity of the nanofluid. In most observed cases, the viscosity was found to increase with increasing nanoparticle concentration and decrease with increasing nanofluid temperature. Different base fluids can affect the viscosity of a nanofluid as well. As the viscosity of the base fluid increases, the viscosity of the nanofluid tends to increase as well [2,23].

Addition of a surfactant may also alter the viscosity of a nanofluid. While many studies have concluded that adding a surfactant will increase the viscosity of the nanofluid; other studies have observed the opposite effect. Qiao et al. [36] observed the change in viscosity with increasing temperature for six different surfactants added to a base fluid of water and graphene nanoparticles. The surfactants used were tested in concentrations of 0.02%, 0.04%, 0.06%, 0.08%, and 0.1% and could be classified into the following three groups: anionic (SDBS, SDS, and PAAS), nonionic (PVP), and cationic (OTAB and CTAB). Qiao et al. [36] found that the addition of surfactant increases the viscosity of the nanofluid. Higher concentrations of surfactant were also correlated with higher viscosity. For example, the addition of PAAS surfactant at a concentration of 0.1% increased the viscosity of the nanofluid by 35.0% at the same temperature [36]. Sayahi and Bahrami [37] examined the effects of adding a SDS surfactant to a Al_2_O_3_-water nanofluid for a pool boiling condition. The addition of surfactant was found to slightly increase the viscosity of the nanofluid when compared to the nanofluid without surfactant.

Khairul et al. [38] observed the effects of adding a SBDS surfactant to both CuO and Al_2_O_3_ water-based nanofluids. The surfactant was added in weight fractions from 0.05% to 0.2% in both nanofluids. The viscosity was highest in both nanofluids when no surfactant was present. With SBDS surfactant concentrations of 0.10% and 0.15%, the viscosity was decreased by an average of 4% and 6%, respectively [38]. However, the concentration of surfactant did not show a clear correlation with viscosity. The Al_2_O_3_-water nanofluid experienced the lowest viscosity at a surfactant concentration of 0.1%, but the CuO-water nanofluid had the lowest viscosity with the 0.15% SBDS concentration [38]. This could be due to the surfactant negatively charging the nanoparticles and causing them to repel one another. The increased particle motion could cause the viscosity to decrease [38].

Additionally, studies by Minakov et al. [39] and Kwek et al. [28] found that decreasing nanoparticle size generally further increases the fluid viscosity. The effect of nanoparticle shape on the viscosity of the nanofluid has typically been found to be insignificant. Zhu et al. [40] investigated the relationship between nanoparticle shape and viscosity within a CuO-dimethicone nanofluid. Wire and spherical-shaped nanoparticles were dispersed in the base fluid and held at a constant temperature of 25 °C. The viscosity increased with increasing volume fraction; however, it was not observed to vary greatly between the differently shaped nanoparticles. Main et al. [41] examined the effects of rod, needle, and spherical-shaped Al_2_O_3_ nanoparticles in ionic liquids. Between the three nanoparticle shapes considered, there was no clear change in viscosity in the respective nanofluids [41].

### 2.3. Effects of Flow Regime on the Heat Transfer Coefficient

**Figure 5:**
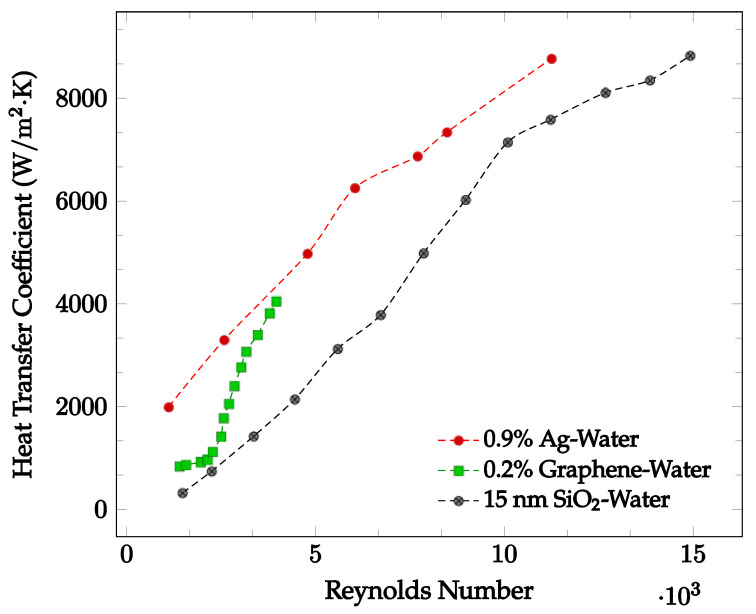
Convective heat transfer coefficient with respect to Reynolds Number over the laminar and turbulent flow regimes for various water-based nanofluids: 0.9% Ag at 48 °C [43], 0.2% Graphene with constant heat flux [5], 15 nm SiO_2_ [44].

The flow regime experienced by the nanofluid may significantly affect the heat transfer coefficient. Demirkir and Erturk [5] found that for a nanofluid traveling through a circular pipe, the heat transfer coefficient increases as the Reynolds number increases, concluding that the heat transfer coefficient increases as the flow regime becomes turbulent. This may be due to an increase in fluid random motion [42]. Godson et al. [43] analyzed the flow of an Ag-water nanofluid with concentrations of 0.3%, 0.6%, and 0.9% through a counter flow heat exchanger. The Ag-water nanofluid convective heat transfer coefficient was found at Reynolds numbers corresponding to three different flow regimes. For Reynolds numbers less than 2500, the flow is characterized to be laminar. For Ag-water nanofluid at 48 °C, the average convective heat transfer coefficient is shown as a function of Reynolds numbers ranging from 1107 to 11,246 below in Figure 5. Here, it is clearly seen that as the Reynolds number increases, the average heat transfer coefficient increases.

Demirkir and Erturk [5] examined the characteristics of a graphene-water nanofluid in transitional flow for volume concentrations of 0.025%, 0.1%, and 0.2%. The nanofluid was pumped through a 2.1 m long copper pipe with an inner diameter of 6 mm and exposed to a constant heat flux. For Reynolds numbers ranging from 1300 to 4000, the average heat transfer coefficient was found. For the nanofluid at a volume concentration of 0.2%, the results are shown in Figure 5. In most cases, as the Reynolds number increases, the average heat transfer coefficient increases due to the increased random motion of nanofluid particles. Zhang et al. [44] analyzed the heat transfer performance of an SiO_2_-water nanofluid within the Reynolds number range of 1000 to 16000 for different diameter nanoparticles at a given concentration. The nanofluid was pumped through a copper tube with length of 2 m, outer diameter of 12 mm, and wall thickness of 1 mm at a constant temperature of 25 °C. For the 15 nm diameter SiO_2_-water nanofluid, the convective heat transfer coefficient as a function of Reynolds number is shown in Figure 5. It was observed that an increase in Reynolds number may result in a convective heat transfer coefficient increase. When Reynolds number increases from 1473 to 15000, the provided experimental data indicated an average heat transfer coefficient increase from 322.1 W/m^2^·K to 8830.3 W/m^2^·K.

Conversely, Liu et al. [45] investigated the change in heat transfer coefficient with varying Reynolds numbers for a Al_2_O_3_-water nanofluid. Over a Reynolds number range of 600 to 4500, the nanofluid experienced laminar, transitional, and fully developed turbulent flow. The heat transfer coefficient did not increase consistently across the Reynolds number range tested. The laminar and fully developed turbulent regions had the greatest heat transfer coefficients, exceeding that of the transition and early turbulent stages.

Generally, the heat transfer coefficient was found to consistently increase as Reynolds number increased. This may be caused by an increase in random fluid motion as Reynolds number increased. However, in some cases it was observed that entering the transitional and early turbulent regions caused a decrease in heat transfer coefficient. As such, the effect of changing flow regime on the heat transfer coefficient is not entirely known.

### 2.4. Optimization of Thermal Conductivity and Viscosity Effects on Heat Transfer Coefficient

The amount of convective heat transfer within a fluid is strongly related to both the viscosity and thermal conductivity of the fluid. A highly viscous fluid would take more pump work to achieve the same fluid velocity than a fluid with lower viscosity. This increased pump work for the same heat transfer output means that the efficiency becomes lower for higher viscosity fluids. As such, it is best to minimize viscosity when trying to maximize the heat transfer of a fluid. Simultaneously, increasing the thermal conductivity increases the heat transfer coefficient. This signifies that maximum heat transfer occurs in fluids of high thermal conductivity and low viscosity. Therefore, in order to engineer a nanofluid with the best heat transfer characteristics, the nanofluid should be optimized to have maximum thermal conductivity and minimum viscosity.

The addition of nanoparticles to a base liquid tends to increase the thermal conductivity of the working fluid. It follows that further increasing the concentration of nanoparticles usually causes a greater increase in the thermal conductivity. Lee et al. [17] and Jang and Choi [16] found that the thermal conductivity of nanofluids tends to increase with increasing nanoparticle concentration. Similarly, viscosity also tends to increase as nanoparticle concentrations increase, as seen in experiments by Sekhar and Sharma [30] and Esfe and Esfandeh [31]. Thus, increasing the nanoparticle concentration has both positive effects, through increasing the thermal conductivity, and negative effects, through increasing the viscosity, on the heat transfer of the fluid. Consequently, the effect of solely increasing the concentration of nanoparticles is likely to differ for different fluid systems.

As the temperature of a nanofluid increases, the thermal conductivity tends to increase as well. Studies by Vajjha and Das [14] and Sundar et al. [15] observed an increase in thermal conductivity with increasing temperature. As such, it was determined that increasing the average temperature of the nanofluid is likely to have a positive effect on the overall heat transfer. Furthermore, increasing the temperature of a nanofluid tends to decrease its viscosity, as observed in experiments by Sharma et al. [34] and Sundar et al. [15]. As such, increasing the temperature of a nanofluid is likely to have a positive effect on the heat transfer coefficient by both increasing the thermal conductivity and decreasing the viscosity of the nanofluid. This also suggests that increasing the temperature of a nanofluid may have a positive effect on the overall heat transfer.

**Figure 6:**
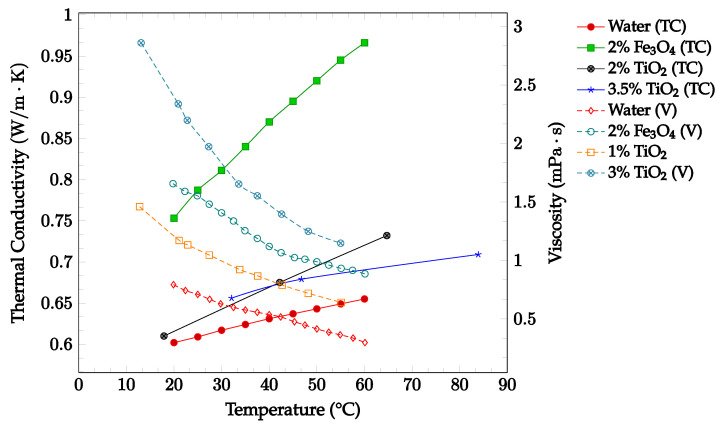
Nanofluid thermal conductivity and viscosity for volume fractions between 0–3.5% and temperatures between 10–90 °C for Fe_3_O_4_-water [15] and TiO_2_-water [46,47,48] nanofluids.

As shown in Figure 6, The effects of changing temperature on the thermal conductivity and viscosity of Fe_3_O_4_-water and TiO_2_-water nanofluids were investigated. Thermal conductivity and viscosity data were gathered for the base fluid and concentrations up to 3.25% in a temperature range of 10–70 °C, as shown below in Figure 6. The data confirmed that an increase in temperature correlated with an increase in thermal conductivity and a decrease in viscosity. The data additionally confirmed that increasing the nanoparticle concentration was correlated with increases in both thermal conductivity and viscosity. These findings reinforce the idea that increasing temperature is likely to have a net positive effect on the overall nanofluid heat transfer coefficient, while increasing concentration is likely to have both positive and negative effects on the nanofluid heat transfer coefficient.

**Figure 7:**
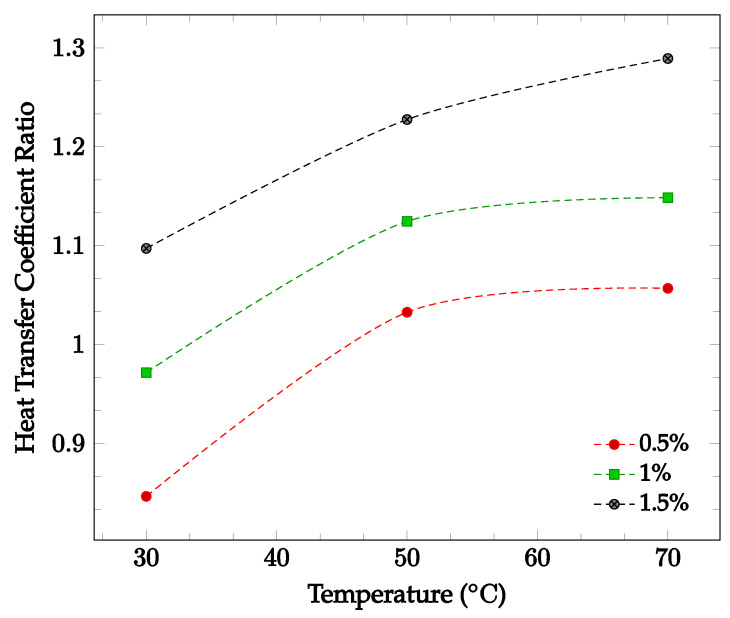
Nanofluid heat transfer coefficient as a function of temperature for volume fractions between 0.5–1.5% of TiO_2_ nanoparticles in 60%:40% EG-water [49].

The effects of changing nanoparticle concentration and fluid temperature directly on the nanofluid heat transfer coefficient were also investigated. Figure 7 shows the effect of changing concentration and temperature on the heat transfer coefficient of a TiO_2_-60:40 EG-water nanofluid. Average heat transfer coefficients were found for concentrations between 0.5% and 1.5% in a temperature range of 30–70 °C. The data gathered by Hamid et al. [49]. found that in this instance, increasing either temperature or concentration led to an increase in overall nanofluid heat transfer coefficient. This supports the claim that increasing nanoparticle concentration may or may not improve the overall heat transfer coefficient, while increasing temperature is likely to increase the heat transfer coefficient.

Most of the experimental data indicated that the nanofluid heat transfer coefficient is dependent on many factors. These factors include choice of base liquid, operating temperature, and nanoparticle concentration and characteristics. It was observed that while each of these parameters may affect the heat transfer coefficient significantly, the effect of these parameters is sometimes small enough to be negligible. As such, nanofluids must be engineered for specific applications in order to achieve the desired nanofluid characteristics. This paper is focused on optimizing the effects of nanoparticle concentration on the nanofluid heat transfer coefficient. In most cases, it was found that increasing nanoparticle concentration led to a simultaneous increase in thermal conductivity and viscosity. As mentioned previously, thermal conductivity enhancement increases the energy transfer inside of the working fluid, leading to an increase in the heat transfer coefficient. Viscosity enhancement leads to a decrease in random nanoparticle motion, decreasing both the energy transfer between fluid layers and the heat transfer coefficient. As such, when increasing the concentration of nanoparticles without making any other changes to the nanofluid, the heat transfer coefficient may increase, decrease, or see little change. If the thermal conductivity enhancement is the dominant effect of increasing nanoparticle concentration, the heat transfer coefficient would increase. However, if the viscosity enhancement is the dominant effect, the heat transfer coefficient would decrease. If neither thermal conductivity nor viscosity enhancement are particularly dominant, little to no change in heat transfer coefficient would likely be observed. This phenomenon is shown in Figure 7, in which it can be seen that increasing nanoparticle concentration in a nanofluid does not always lead to enhanced a heat transfer coefficient. In order to optimize the heat transfer coefficient, it is desired to increase nanofluid thermal conductivity while simultaneously decreasing viscosity. Experimental data indicated that nanofluid thermal conductivity tends to increase with both increasing nanoparticle concentration and temperature. However, it was observed that nanofluid viscosity tends to decrease with increasing temperature. Therefore, by increasing operating temperature, it is possible to enhance nanofluid thermal conductivity while simultaneously reducing the negative impact of viscosity enhancement on the heat transfer coefficient. Figure 6 and Figure 7 show that nanofluid thermal conductivity increases with both increasing nanoparticle concentration and temperature, while viscosity decreases with increasing temperature. As such, it may be possible to reduce or even eliminate the negative effect of viscosity on the heat transfer coefficient by increasing operating temperature. The data shown in Figure 7 observed an increasing nanofluid heat transfer coefficient for constant nanoparticle volume fraction with increasing temperature, agreeing with this theory. A relatively high operating temperature is common in applications for cooling systems or thermal management of electronic devices, as they typically operate at high fluid temperatures.

## 3. Experimental Setup

**Figure 8:**
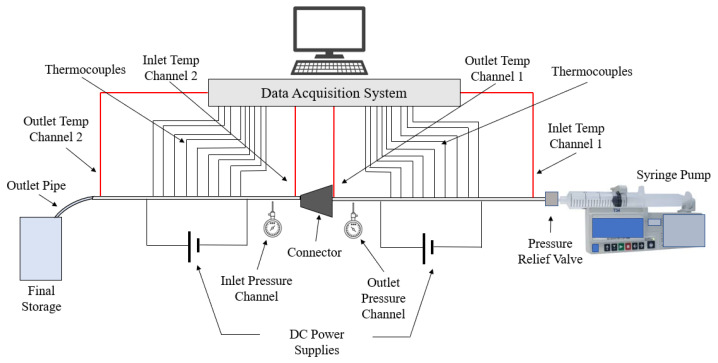
Setup used to measure the heat transfer coefficient along two microchannels connected in series.

**Figure 9:**
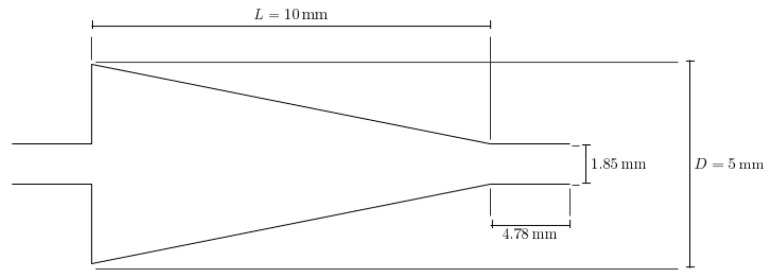
Detailed geometry and dimensions of the nozzle connector used. The same nozzle was used in both converging and diverging configurations.

This paper seeks to determine how the heat transfer between two microchannels are affected by the addition of a nozzle-shaped connector. The setup used to measure the heat transfer coefficient across both microchannels is shown below in Figure 8 and features a system of two pipes in series. The nanofluid is non-circulating and exits into a final storage container, instead of cycling back to the first channel. Pumping power is provided via a New Era System NE-8000 pump fitted with a Hamilton 100 mL syringe. The syringe is connected to the first channel by use of Hamilton 86510 plastic tubing inserted into an Upchurch Scientific 53500-816 union, and the exit of the second channel is connected to the same tubing and union. Within these unions, Omega 5TC-TT-K-30-36 thermocouples are placed to measure the temperature of the fluid at the inlet and exit of the setup. The microchannels themselves each have inner and outer diameters of 210 μm and 413 μm, respectively. The nozzle within the connector is 10 mm in length, with the small and large diameters having a size of 1.8542 mm and 3 mm, respectively, as shown in Figure 9. The nozzle is used in both diverging and converging configurations to determine the effect of each setup on the heat transfer coefficient within the system. The nozzle is connected to both channels by use of Upchurch Scientific 53500-816 unions on either side. In order to directly measure the fluid temperature before and after the nozzle, thermocouples are placed inside the unions.

A Sorensen XPH 20-20 DC power supply is used to send a direct current through each microchannel, which are wired in parallel at the ends of each pipe. The pipes are then heated by conversion from electrical to thermal energy due to the resistance of the steel. The power supply is set such that the temperature at the outlet was 80 °C. As a result, a constant heat flux is produced within the channels. Additionally, the power supply can be set between 0–20 V and 0–20 A DC as needed. Assuming that all of the electrical energy dissipated is converted to thermal energy, the law of conservation of energy is able to be used to determine the heat generated in each channel.

The temperature at each point on the channels are measured using RS Pro 397-1589 thermocouples, with a reported error of ±1.5 °C. Each of the thermocouples was connected to the channel through use of thermally conductive Duralco 132 resin and hardener. After attachment of the thermocouples, each channel was covered in a layer of nonconductive 3M Scotch-Weld Epoxy Adhesive 2214 to prevent heat loss outside of the pipes from causing erroneous measurement. Each channel was then covered by 1 cm dry insulation to further reduce heat loss. The pressure in the channels is measured at the inlet and outlet of each pipe by use of Omega Engineering PX26-005GV pressure transducers, powered by an Omega PST 4130 power supply. The pressure sensors used have a reported maximum error of ±1%.

The temperature, pressure, and electrical data is collected through use of a National Instruments NI cDAQ-9178 data acquisition system. The thermocouples are connected to two NI 9213 cards, giving each channel a separate card. The pressure transducers are connected to one NI 9218 card using NI 9982 adapters and are used to measure the pressure drop across the connector. The voltage drop across the system is measured using an NI 9221 card. All data received from the DAQ system was monitored and collected through National Instruments LabVIEW software.

## 4. Materials and Methods

### 4.1. Nanofluid Synthesis

Looking at literature examined [50] and our previous experiences [29], it was found that Fe_3_O_4_-water nanofluid would work best in the present experiment. The experiment was conducted using 99.5% pure Fe_3_O_4_ nanoparticles purchased from US Research Nanomaterials, Inc, Houston, TX, USA. The particles were listed as having spherical morphology and an average particle size of 15 to 20 nm. The nanofluid was formulated by mixing the Fe_3_O_4_ nanoparticles in deionized water with 1.0% concentration by weight, before it was sonicated for thirty minutes to ensure the suspension was well-dispersed. The necessary physical properties of the nanofluid, namely density, specific heat, and volume fraction, were calculated using Equations (1) through (3), as given by Sundar et al. [15]:

(1) $$\rho_{nf}=\phi \rho_{p}+\left(1-\phi \right)\rho_{bf}$$

(2) $$c_{nf}=\frac{\phi \left(\rho c\right)+\left(1-\phi \right){\left(\rho c\right)}_{bf}}{\rho_{nf}}$$

(3) $$\phi =\frac{w\rho_{bf}}{\rho_{p}\left(1-w\right)+w\rho_{bf}}$$

where ρ is the density in kg/m^3^, ϕ is the nanoparticle volume concentration, c is the specific heat in J/kg·K, and w is the pure weight fraction of the nanofluid. Parameter nomenclature is reviewed in detail in Table A1 of Appendix A. Additionally, in Table 1 below, the important properties of the nanoparticles, base fluid, and nanofluid are specified. These values were determined by Sundar et al. [15] for various temperatures and were used to interpolate for the relevant temperature.

### 4.2. Heat Transfer Measurement

The nanofluid temperatures at the channel inlet and outlet were measured using Omega 5TC-TT-K-30-36 thermocouples as described previously. The mass flow rate was directly configured using the pump. The objective of the present study is to calculate and optimize the heat transfer coefficient in the microchannel system described. The heat transfer coefficient can be calculated by the following equation:

(4) $$h\left(x\right)=\frac{\dot{q}}{\left(T_{s}\left(x\right))-(T_{f}\left(x\right)\right)}$$

where $h\left(x\right)$ is the local heat transfer coefficient at distance x from the inlet, $\dot{q}$ is the heat flux in W/m^2^, and $T_{s}\left(x\right)$ is the measured temperature at the surface of the microchannel at that distance. From this equation, the heat transfer coefficient can be calculated once the heat flux and fluid temperature are known. Using the known inlet and outlet temperatures and mass flow rate and the specific heat as given in Table 1, the heat flux can be determined through use of the following equation:

(5) $$\dot{q}=\frac{\dot{m}c\Delta T}{A_{s}}=\frac{\dot{m}c\left(T_{out}-T_{in}\right)}{A_{s}}$$

where $\dot{m}$ is the mass flow rate in kg/s, $\Delta T=T_{out}-T_{in}$ is the difference between outlet and inlet temperatures in K, and $A_{s}$ is the surface area through which heat is being transferred in m^2^. Through use of the known inlet temperature, the fluid temperature at any distance from the inlet can be found from the following relationship:

(6) $$T_{f}\left(x\right)=T_{in}+\frac{\dot{q}\pi D}{c_{p}\dot{m}}x$$

where $T_{f}\left(x\right)$ is the fluid temperature at distance x from the inlet and D is the microchannel diameter. All measurements were taken after the nanofluid reached steady state flow. For the given conditions, the heat transfer coefficient was calculated as a function of position. The local temperatures and heat transfer coefficients at each point measured were recorded at a frequency of 5 Hz. The data was aggregated by position. The samples accumulated at each position was compared over time, and the averages and standard deviations of the heat transfer coefficient at each point were determined. The standard deviations for each point were found to be very small in comparison to the value of the heat transfer coefficient, so the average value was taken without use of error bars. Similar results were found by Rea et al. [51] and Vafaei et al. [29].

## 5. Results and Discussion

The characteristics and concentration of nanoparticles have significant effects on the heat transfer properties of the resulting nanofluid, including the thermal conductivity, viscosity, and heat transfer coefficient [29]. As shown in Figure 1, nanoparticle inclusion tends to increase the thermal conductivity relative to the base fluid. This increase in thermal conductivity is likely due to the high thermal conductivity of the particles used compared to that of traditional base fluids. Additionally, the increase in thermal conductivity may be due to the increased energy transport between fluid layers driven by random particle motion. Increasing fluid temperature was also found to increase nanofluid thermal conductivity, as higher temperatures tend to increase random particle motion and cause more frequent particle collisions. Furthermore, as seen in Figure 2, increasing the concentration of nanoparticles tends to increase the thermal conductivity of the resultant nanofluid. This trend was found to hold for nanofluids of varying base fluid and nanoparticle materials with varying effectiveness, but it was observed that the thermal conductivity may decrease at high enough concentrations. This may be due to particle agglomeration at high concentrations [29]. It was found that that base fluids with higher thermal conductivities caused nanofluids with higher thermal conductivities. It was seen that nanoparticles with a high aspect ratio and lower sphericity tended to lead to nanofluids with particularly high thermal conductivities [26,29]. Additionally, decreasing nanoparticle size was shown to generally increase nanofluid thermal conductivity. Both of these observations may be attributed to an increase in the surface area to volume ratio as either the aspect ratio increases or size decreases. In cases where a surfactant was present, it was sometimes observed that high concentrations of surfactant would lead to the nanoparticles falling out of suspension, agglomerating, and lowering the overall thermal conductivity of the nanofluid [23,24,25]. The exact points at which agglomeration may occur was found to vary per nanofluid, requiring proper optimization to achieve the desired results.

The viscosity of fluids was also found to change upon the addition of nanoparticles. As shown in Figure 3, adding nanoparticles to a fluid typically was observed to increase the fluid’s viscosity. Furthermore, increasing the nanoparticle concentration generally further increased the viscosity. Conversely, nanofluid viscosity was found to decrease as the temperature of the fluid was increased, as visible in Figure 4. This phenomenon was found to occur for nanoparticles of various compositions and concentrations. The loss of viscosity at higher temperatures may be caused by intermolecular forces becoming weaker with an increase in temperature. Changing the base fluid was found to change the viscosity of the nanofluid, with higher viscosity base fluids being correlated with high viscosity nanofluids. Increasing nanoparticle size was found to increase as nanoparticle size was decreased. This may be due to the higher tendency of smaller particles to agglomerate together, increasing the viscosity [2]. Changing nanoparticle shape was not observed to have any significant effect on the nanofluid viscosity. Additionally, the effect of surfactant use on viscosity was investigated. Some studies found that surfactant use increased the nanofluid viscosity [36,37], while another found that viscosity decreased [38]. Khairul et al. [38] posed that the surfactant could give the nanoparticles a slight negative charge, driving repulsion and decreasing the viscosity by increasing the particle motion.

Nanoparticles have a significant impact on convective heat transfer coefficient. The increase in the heat transfer coefficient as concentration is increased may be caused by both the higher thermal conductivity and an increase in chaotic movement of nanoparticles. However, the increase in viscosity associated with higher concentrations of nanoparticles can cause the heat transfer coefficient to decrease when concentration is increased. As such, the effect of increasing concentration was not strictly an increase in the heat transfer coefficient. Additionally, at high enough concentrations, there is a risk of depositing nanoparticles on pipe walls [52]. Nanofluids using cylindrical nanoparticles with high aspect ratios were found to have higher heat transfer coefficients than identical nanofluids using spherical or low aspect ratio nanoparticles [3,4]. This may be due to the higher surface area to volume ratio or the increased contact between nanoparticles facilitating more energy transfer within the fluid. The prevention of particle agglomeration caused by surfactant addition into nanofluids may increase the nanofluid heat transfer coefficient [36], but surfactants have also been observed to increase nanofluid viscosity sometimes, which can lead to a lower heat transfer coefficient [17]. Additionally, as seen in Figure 5, the heat transfer coefficient was found to generally increase as the Reynolds number increased. This may be due to an increase in the amount of random particle motion [5]. The effects of base liquid, possible surfactant, concentration, and characteristics of nanoparticles (size, shape, and material) on thermal conductivity, viscosity and heat transfer coefficient were explained in detail in references [29,33,42].

**Figure 10:**
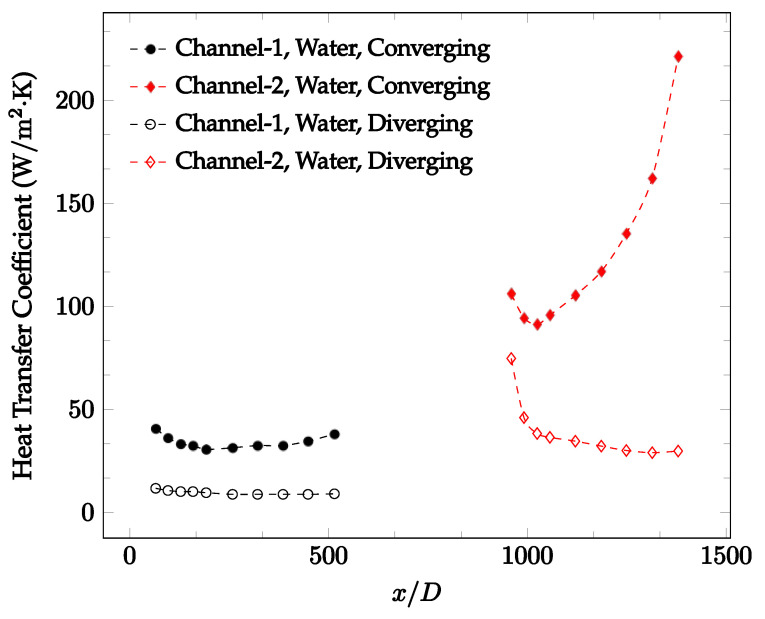
Change in convective heat transfer coefficient of deionized water as a function of dimensionless distance for a Reynolds numbers of 25. Converging and diverging nozzles are used as connectors.

**Figure 11:**
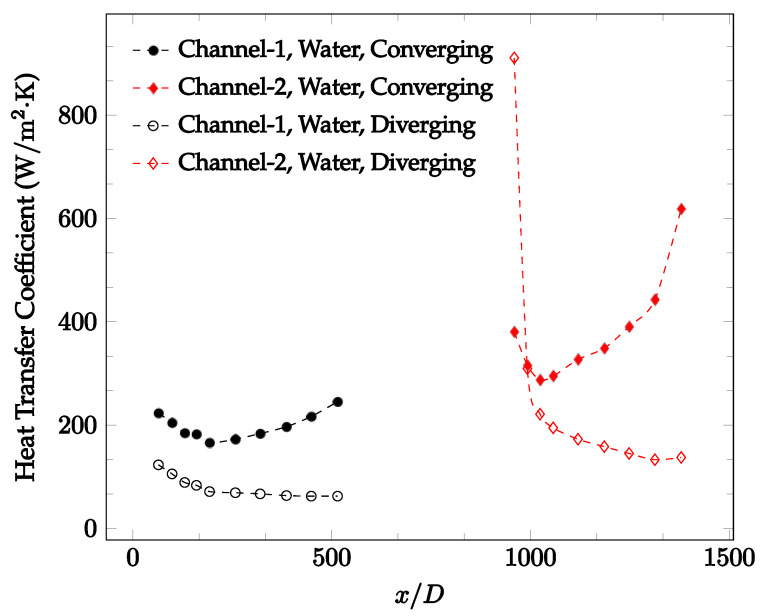
Change in convective heat transfer coefficient of deionized water as a function of dimensionless distance for a Reynolds number of 50. Converging and diverging nozzles are used as connectors.

**Figure 12:**
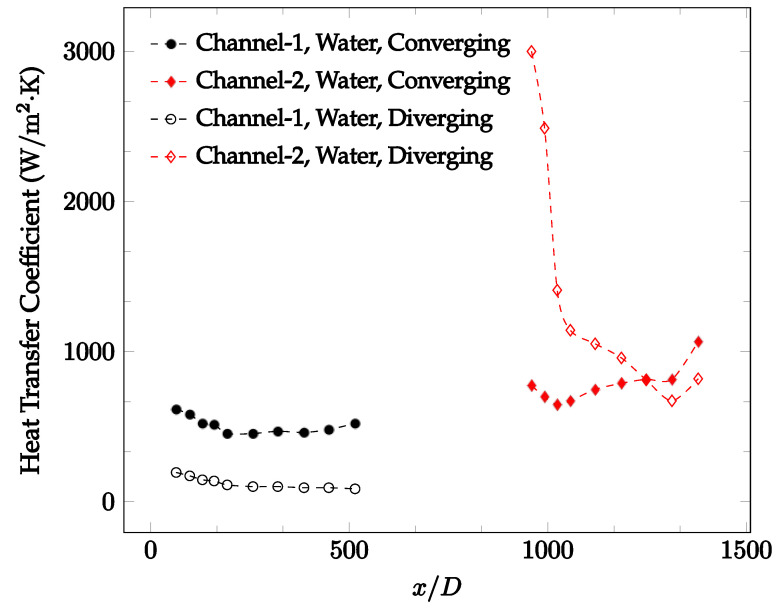
Change in convective heat transfer coefficient of deionized water as a function of dimensionless distance for a Reynolds number of 100. Converging and diverging nozzles are used as connectors.

**Figure 13:**
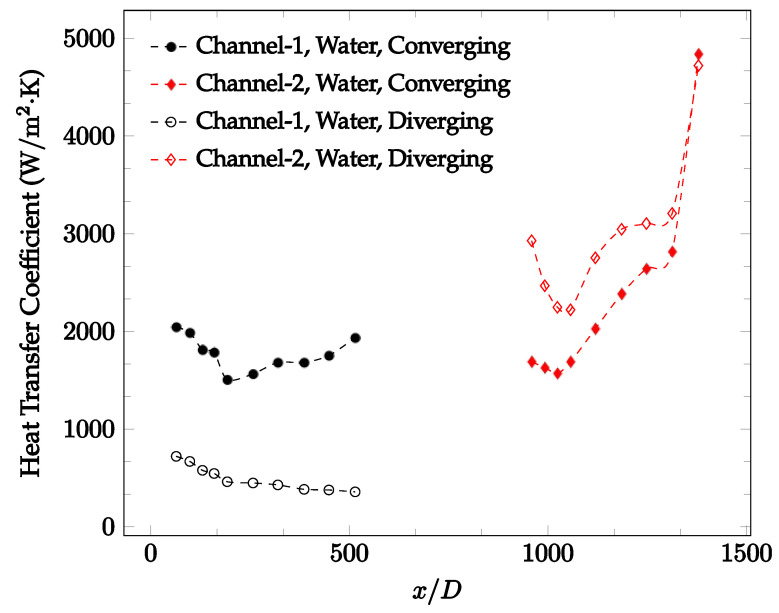
Change in convective heat transfer coefficient of deionized water as a function of dimensionless distance for a Reynolds number of 200. Converging and diverging nozzles are used as connectors.

**Figure 14:**
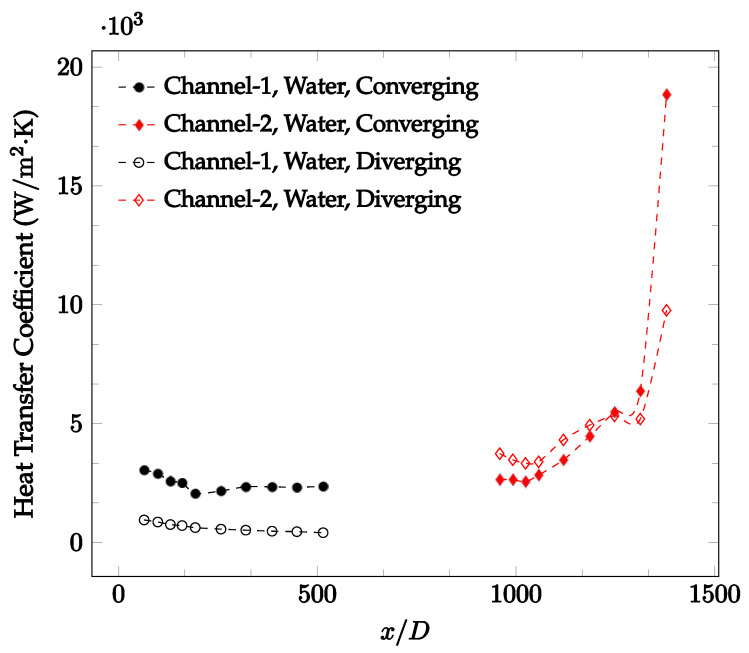
Change in convective heat transfer coefficient of deionized water as a function of dimensionless distance for a Reynolds number of 300. Converging and diverging nozzles are used as connectors.

**Figure 15:**
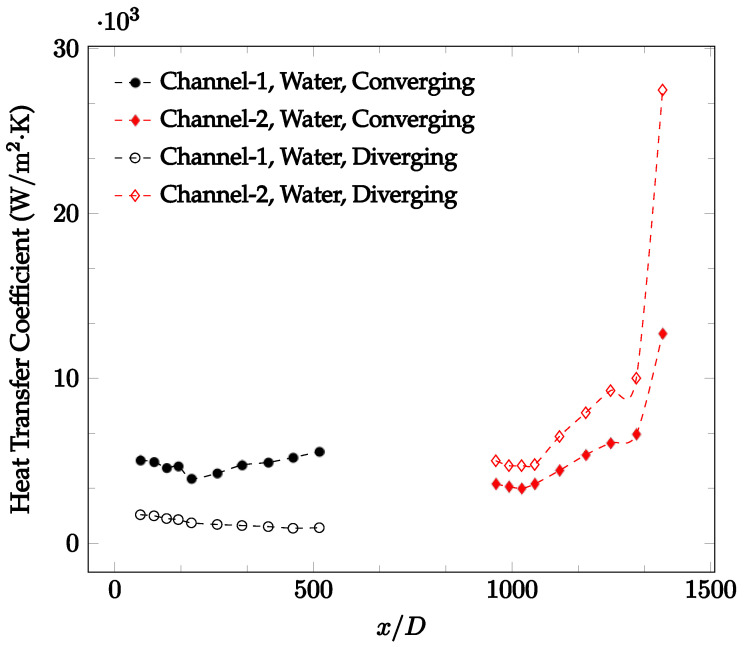
Change in convective heat transfer coefficient of deionized water as a function of dimensionless distance for a Reynolds number of 400. Converging and diverging nozzles are used as connectors.

The forced convection heat transfer coefficient was measured inside the microchannels as a function of distance. This paper seeks to answer the questions of how the addition of nanoparticles in the base fluid impacts the heat transfer coefficient, how changing the Reynolds number impacts the heat transfer coefficient, and how changing the geometry of the connector affects the heat transfer coefficient. To answer these questions, forced convection heat transfer coefficient was measured as a function of distance for deionized water and deionized water–Fe₃O₄ nanofluids using different connectors. The concentration of the water–Fe₃O₄ nanofluids was 1 wt% and the range of the Reynolds number inside the microchannel was 25–400. Three connectors with varying geometry were used between two serial microchannels. The first was a spherical connector for which the diameter was 10 mm. Meanwhile, the second and third connectors were converging and diverging nozzles. Figure 10, Figure 11, Figure 12, Figure 13, Figure 14 and Figure 15 show the heat transfer coefficient as a function of distance for deionized water inside both microchannels when Re = 25, 50, 100, 200, 300, and 400, respectively. Here, converging and diverging nozzles are used as connectors. Error bars are removed from all figures due to their significantly small size.

It was observed that heat transfer coefficient was relatively higher in the second microchannel because the level of random motion of the molecules increases inside the connector. It was also observed that the heat transfer coefficient decreases in the first channel smoothly as *x* increases [42]. Similarly, the heat transfer coefficient in the second channel starts at a local maximum, then decreases with increasing x.

It was observed that the heat transfer coefficient increases at the end of the second microchannel, in some cases (see Figure 10, Figure 11 and Figure 12). The enhancement of the heat transfer coefficient at the end of the second channel might be related to the reduction of fluid viscosity, because of the higher fluid temperature at the end of channel. The viscosity decreases with fluid temperature, and consequently, the random motion of molecules increases. This phenomenon is more effective at low Reynolds numbers where the level of randomness of molecules is relatively low. At a high Reynolds number, the level of randomness of molecules is high enough such that this phenomenon is not effective. This is because the level of randomness of molecules increases with Reynolds number. However, this phenomenon was not observed when the diverging connector was used. In the case of the diverging connector, the outlet temperature at the second microchannel was relatively low because heat transfer coefficient was relatively low, and consequently, this phenomenon was not observed.

**Figure 16:**
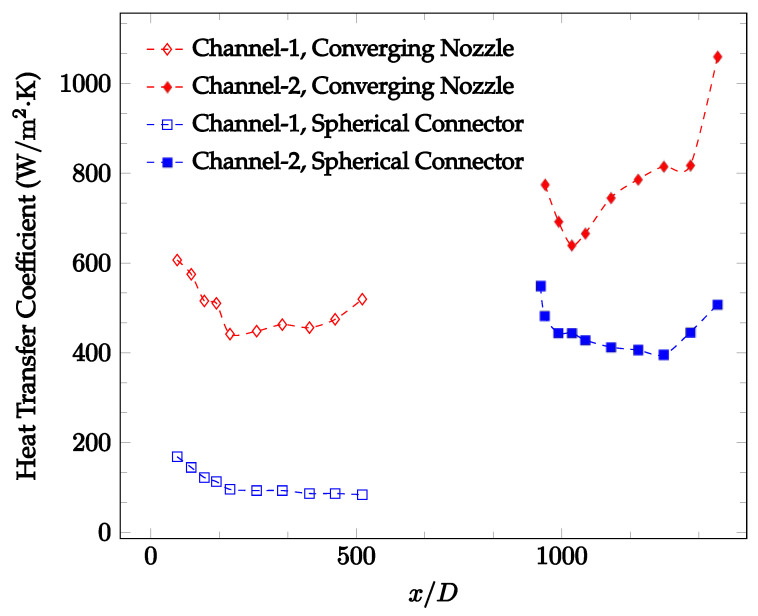
Change in convective heat transfer coefficient of deionized water as a function of dimensionless distance for a Reynolds number of 100. Converging and spherical connectors are used.

Figure 10, Figure 11 and Figure 12 shows that at low Reynolds numbers, the level of the heat transfer coefficient in the second microchannel is higher when the converging connector is used compared to the diverging connector. As observed in Figure 13, Figure 14 and Figure 15, as the Reynolds number increases, the level of the heat transfer coefficient in the second microchannel decreases when the converging connector is used compared to the diverging connector. Eventually, the level of the heat transfer coefficient in the second microchannel is higher when the diverging connector is used compared to the converging connector. As fluid is moving into the converging connector, the bulk velocity increases because of conservation of mass; therefore, the random motion of molecules increases as fluid passes through. The effect of this phenomenon is significant when Reynolds number is low. As Reynolds number increases, level of randomness of molecules increases and the effect of this phenomenon becomes negligible. In addition, at high Reynolds numbers, the fluid impacts the vertical wall at the end of connector and thus increases the level of randomness of molecules before leaving the connector (see Figure 13, Figure 14 and Figure 15). Across the range of Reynolds numbers tested, the converging nozzle was generally found to cause a higher heat transfer coefficient in the first microchannel, with results in the second microchannel varying. As such, the converging nozzle was typically found to have a higher heat transfer coefficient overall. As seen in Figure 16, the converging nozzle was also found to have a higher heat transfer coefficient when compared to the spherical connector.

**Figure 17:**
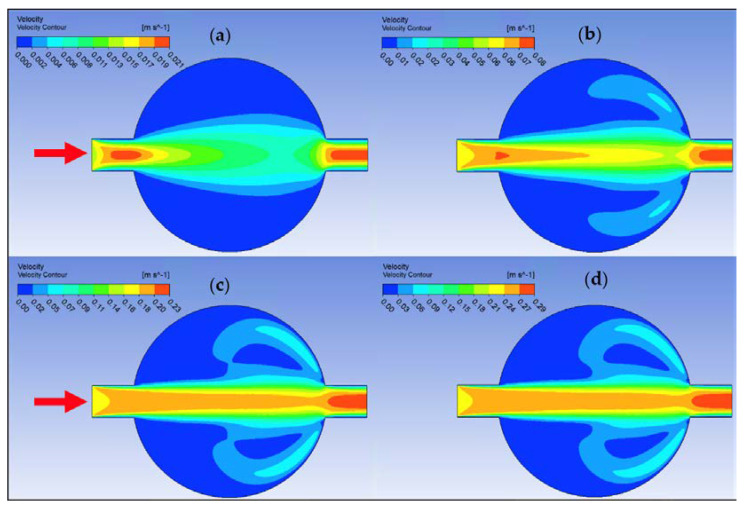
Velocity contour for deionized water in spherical connector. (**a**) Re = 25, (**b**) Re = 100, (**c**) Re = 300, and (**d**) Re = 400.

**Figure 18:**
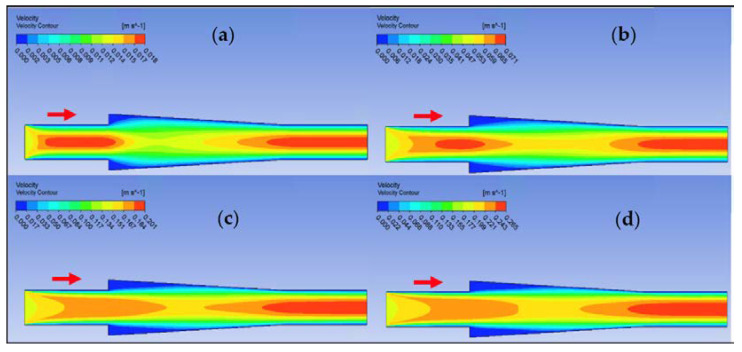
Velocity contour for deionized water in converging connector. (**a**) Re = 25, (**b**) Re = 100, (**c**) Re = 300, and (**d**) Re = 400.

**Figure 19:**
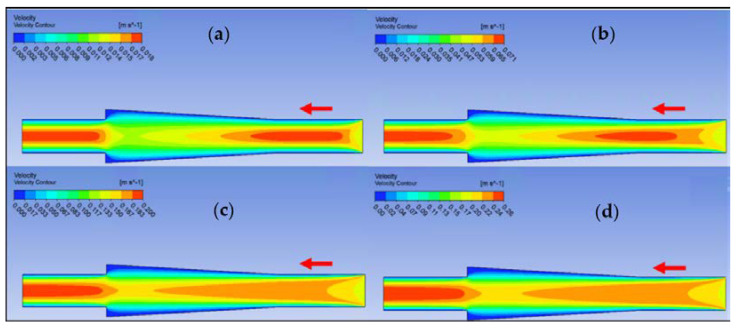
Velocity contour for deionized water in diverging connector. (**a**) Re = 25, (**b**) Re = 100, (**c**) Re = 300, and (**d**) Re = 400.

Ansys Fluent software was used to conduct computational fluid dynamics (CFD) calculations. The continuity and Navier–Stokes equations were solved numerically to predict the velocity distribution and plot velocity contours in three different connectors. The geometry of connectors can be seen in Figure 9, and mesh size was small enough to make sure results are independent of mesh size. The inlet velocity and outlet pressure applied as boundary conditions. Figure 17 shows the velocity contours in a spherical connector when the Reynolds number is equal to 25, 100, 300, and 400. This figure shows how velocity inside the spherical connector would change as Reynolds number increases. Figure 18 shows the velocity contours in a converging connector when Reynolds number is equal to 25, 100, 300, and 400. This figure shows how the velocity inside the converging connector would change as Reynolds number increases. Figure 19 shows the velocity contours in a diverging connector when Reynolds number is equal to 25, 100, 300, and 400. This figure shows how the velocity inside the diverging connector would change as Reynolds number increases. Figure 17, Figure 18 and Figure 19 show the variation of velocity contours as a function of Reynolds number inside different connectors. In the case of a spherical connector, fluid moves into the connector like a bullet at low Reynolds numbers. At a high Reynolds number, the working fluid hits the end of the connector and produces two circulations. The intensity of the circulation increases as Reynolds number increases. These circulations can play a significant role on the enhancement of the random motion of molecules at the outlet of the connector. In the case of the diverging connector, the bulk velocity of the working fluid decreases as the fluid passes through, and this phenomenon is visible in high and low Reynolds numbers. Bulk velocity inside a connector increases, as Reynolds number increases. It is obvious that in the case of the converging connector, bulk velocity of working fluid increases as fluid goes through, and consequently the random motion of molecules increases. In all three connectors, the fluid moves into the connector like a bullet and produces collisions with the molecules of the working fluid inside the connector. Therefore, the level of random motion of the molecules increases at the outlet of the connectors.

**Figure 20:**
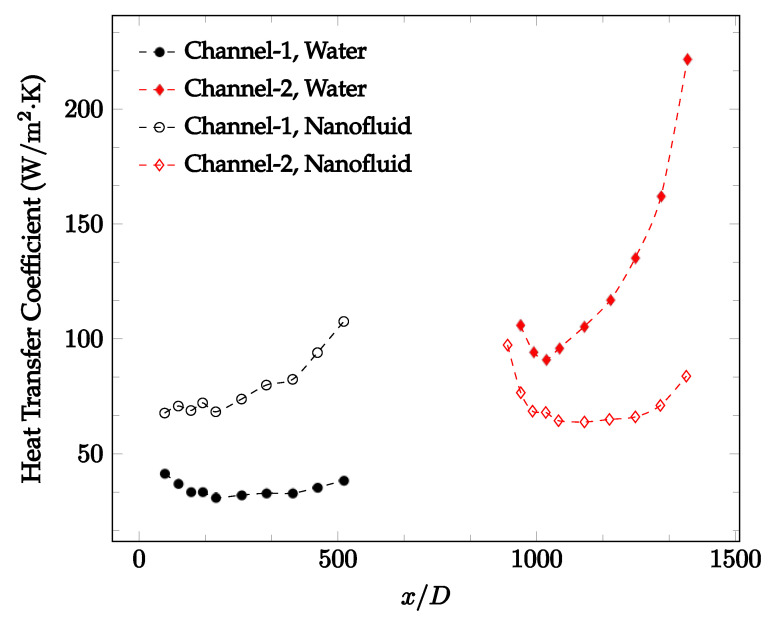
Change in convective heat transfer coefficient of deionized water and Fe_3_O_4_-deionized water nanofluids as a function of dimensionless distance for a Reynolds number of 20–25. The nanoparticle concentration was 1% by weight. A converging nozzle is used as a connector.

**Figure 21:**
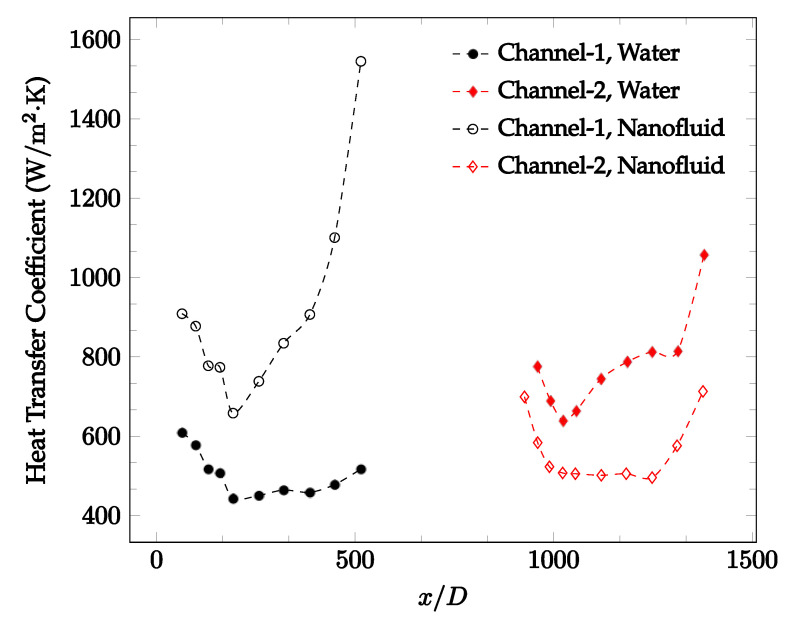
Change in convective heat transfer coefficient of deionized water and Fe_3_O_4_-deionized water nanofluids as a function of dimensionless distance for a Reynolds number of 100–114. The nanoparticle concentration was 1% by weight. A converging nozzle is used as a connector.

**Figure 22:**
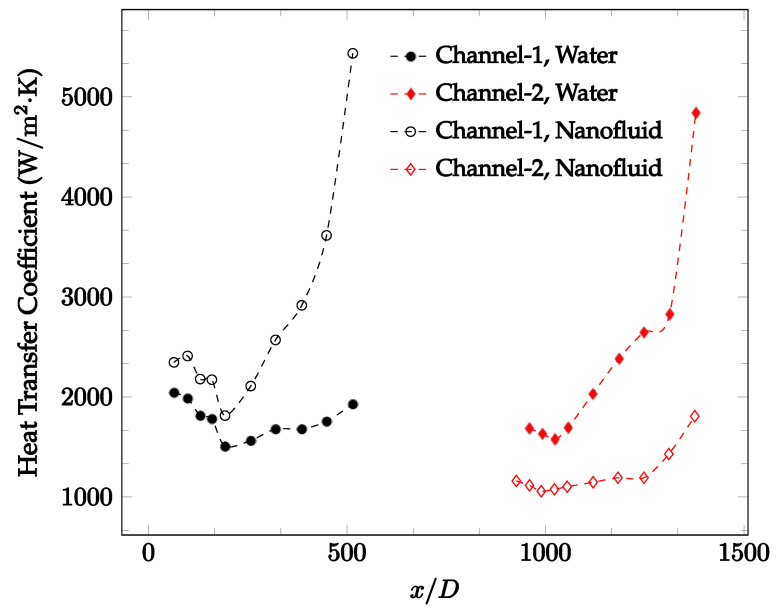
Change in convective heat transfer coefficient of deionized water and Fe_3_O_4_-deionized water nanofluids as a function of dimensionless distance for a Reynolds number of 200–236. The nanoparticle concentration was 1% by weight. A converging nozzle is used as a connector.

**Figure 23:**
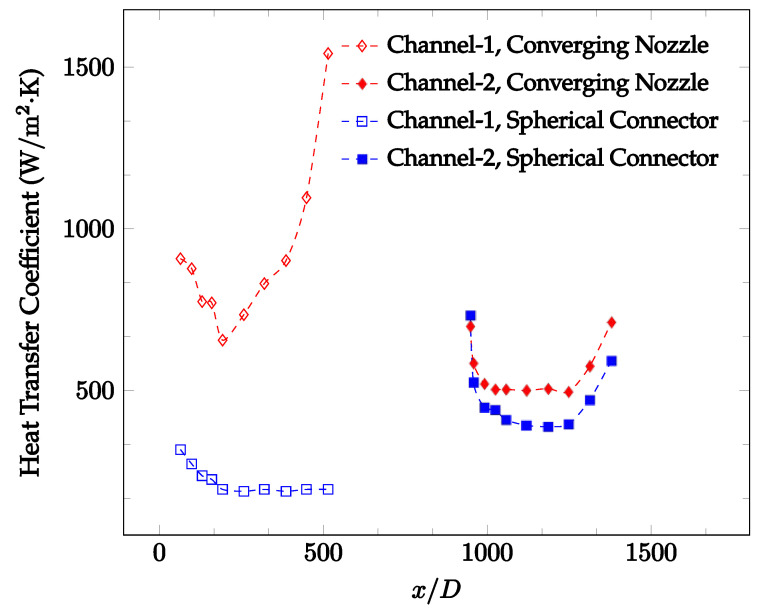
Change in convective heat transfer coefficient of Fe_3_O_4_-deionized water nanofluid as a function of dimensionless distance for a Reynolds number of 100–114. The nanoparticle concentration was 1% by weight. Converging and spherical connectors are used.

Figure 20, Figure 21, Figure 22 and Figure 23 show variation of the heat transfer coefficient as a function of location for deionized water and 1 wt% Fe_3_O_4_-deionized water nanofluids when Reynolds number was in a range of 25–236. The concentration of Fe_3_O_4_-deionized water nanofluid was 1 wt% and the connector between microchannels was a converging nozzle. These figures consistently show that heat transfer coefficient in the first microchannel is higher when the working fluid is 1 wt% Fe_3_O_4_-deionized water nanofluid, but the heat transfer coefficient in the second microchannel is higher when the working fluid is deionized water. Obviously, the thermal conductivity of 1 wt% Fe_3_O_4_-deionized water nanofluid is higher than that of deionized water. Therefore, the forced convection heat transfer coefficient in the first microchannel is higher when the working fluid is 1 wt% Fe_3_O_4_-deionized water nanofluid. The impact of the converging connector on the heat transfer coefficient in the second microchannel was negligible, which might be related to the comparatively higher density of nanofluid inside the converging connector. According to the continuity equation, as the density of working fluid increases, the velocity decreases for a given cross sectional area inside the converging connector. Furthermore, the random motion of molecules and nanoparticles decreases as bulk velocity decreases inside the converging nozzle. Consequently, the heat transfer coefficient decreases in the second microchannel. Figure 20, Figure 21 and Figure 22 show the variation of the heat transfer coefficient as a function of location for 1 wt% Fe_3_O_4_-deionized water nanofluids. The Reynolds number was in the range of 20–236 and the concentration of Fe_3_O_4_-deionized water nanofluid was 1 wt%. This figure compares the heat transfer coefficient inside the first and second microchannels for the converging nozzle and spherical connectors. Like Figure 10, Figure 11, Figure 12, Figure 13, Figure 14 and Figure 15, Figure 20, Figure 21 and Figure 22 show that heat transfer coefficient is higher in both microchannels when the connector is a converging nozzle.

## 6. Conclusions

In this paper, a review was carried out on the effects of nanoparticles on the thermal conductivity and viscosity of nanofluids. It was found that, in the majority of cases, nanoparticle loading simultaneously increased the thermal conductivity and viscosity of the working fluid. These two effects then drive the observed changes in the heat transfer coefficient. Specifically, the increase in the thermal conductivity results in an increase in the heat transfer coefficient. However, the increase in the viscosity decreases the heat transfer coefficient, due to the lower random motion of molecules and nanoparticle and consequently, smaller amount of energy transfer between fluid layers. Therefore, the heat transfer coefficient will increase only when the effect of the thermal conductivity is dominant and will decrease only when the effect of viscosity is dominant.

The effect of the geometry of the connector between two serial stainless-steel microchannels on the second channel’s convective heat transfer coefficient was investigated. Factors considered include the Reynolds number, working fluid, and connector shape. It was found that connector would allow the working fluid to refresh its memory before entering the second microchannels, raising the heat transfer coefficient in the second microchannel. As such, the heat transfer coefficient in the second channel starts from a local, and occasionally absolute, maximum. Then, there is a decrease as the distance from the microchannel entrance increases. This increase at the beginning of the second microchannel causes the average heat transfer coefficient across both microchannels to increase. The connectors also have a great potential to increase the level of random motion of molecules and nanoparticles before the entrance into the second microchannel. This may contribute to enhancement of the heat transfer coefficient in the second channel. However, this enhancement was observed at lower Reynolds numbers. The random motion enhancement due to the connector is more significant at sufficiently small Reynolds numbers. In general, it was observed that adding a connector between two microchannel would increase the heat transfer coefficient significantly.

CFD calculations are conducted to understand the effects of Reynolds number and the shape of the connector on the fluid flow inside the connector. The CFD calculations indicated that the Reynolds number and the shape of the connector play a significant role on the fluid flow and random motion of molecules inside the connector, and consequently, the heat transfer coefficient inside the second microchannel. CFD calculations were necessary to understand the flow motion inside different connectors.

The effect of Fe_3_O_4_ nanoparticle addition to deionized water on heat transfer coefficient was investigated in both microchannels. It was seen that the Fe_3_O_4_-water nanofluid typically had higher heat transfer coefficients than the pure deionized water in the first microchannel. This may be due to the high thermal conductivity of the Fe_3_O_4_-water nanofluid relative to that of deionized water. Additionally, the addition of nanoparticles is likely to increase the energy transfer between fluid layers as well as the level of random motion. The heat transfer coefficient of the nanofluid was found to decrease in the second microchannel relative to that of deionized water. This effect may be caused by the higher density of the nanofluid inside of the converging connector. Based on the continuity equation, as the density of working fluid increases, the velocity decreases for a given cross sectional area inside the converging connector. Obviously, the random motion of molecules and nanoparticles decreases as bulk velocity decreases inside the converging nozzle, and consequently, the heat transfer coefficient decreases in the second microchannel.

This research paper indicated that adding a connector between microchannel has a great potential to enhance heat transfer coefficient and provide an opportunity to make smaller and more powerful electronic devices on earth and space.

## Figures and Tables

**Table 1:** List of relevant physical properties, including density, specific heat, dynamic viscosity, and thermal conductivity, for the nanofluid utilized.

Material	Density [kg/m^3^]	Specific Heat [J/kg · K]	Dynamic Viscosity [mPa · s]	Thermal Conductivity [W/m · K]
Fe_3_O_4_ [15]	5810	670	-	-
Water ^1^ [15]	986.7	4182	0.523	0.652
1 wt% Fe_3_O_4_-water ^1^ [15]	996.23	4174.10	0.41	0.7450

^1^ For T_avg_ = 52.5 °C.

## Data Availability

The data presented in this study are available on request from the corresponding author.

## Appendix A

**Table A1:** Nomenclature with standard units.

Symbol	Definition	Units
k	Thermal conductivity	(W/m·K)
μ	Dynamic viscosity	(kg/m·s)
ρ	Density	(kg/m^3^)
ϕ	Volume concentration of nanoparticles	(-)
c	Specific heat	(J/kg·K)
w	Weight concentration of nanoparticles	(-)
$\dot{q}$	Heat flux	(W/m^2^)
$\dot{m}$	Mass flow rate	(kg/s)
T	Temperature	(K)
$A_{s}$	Surface area	(m^2^)
D	Microchannel diameter	(m)
x	Distance from inlet	(m)
h	Heat transfer coefficient	(W/m^2^·K)
Subscripts		
nf	Nanofluid	
p	Nanoparticle	
bf	Base fluid	
f	Fluid	
s	Surface	
